# Biochemical Diagnosis of Phaeochromocytoma and Paraganglioma in Children and Adolescents: A Retrospective Cohort Study

**DOI:** 10.1111/cen.70025

**Published:** 2025-08-21

**Authors:** Kristin Potthoff, Tamara Prodanov, Lara M. Knigge, Angela Hübner, Stefan Bornstein, Jacques Lenders, Karel Pacak, Graeme Eisenhofer, Christina Pamporaki

**Affiliations:** ^1^ Department of Medicine ΙΙI University Hospital Carl Gustav Carus at the TU Dresden Dresden Germany; ^2^ National Institutes of Health (NIH) Bethesda USA; ^3^ Department of Paediatrics University Hospital Carl Gustav Carus at the TU Dresden Dresden Germany; ^4^ Department of Internal Medicine Radboud University Medical Center Nijmegen the Netherlands

**Keywords:** children, diagnostic performance, metanephrine, normetanephrine, paraganglioma, phaeochromocytoma

## Abstract

**Introduction:**

Currently, it is unclear whether plasma free or 24‐h urinary fractionated metanephrines are preferable for diagnosis of phaeochromocytoma/paraganglioma (PPGL) in children.

**Objectives:**

To investigate whether measurements of plasma free and 24‐h urinary fractionated metanephrines are reliable tests for screening for PPGL in children.

**Methods:**

This retrospective study included a cohort of 138 paediatric patients (5 to 18 years), 64 with and 74 without PPGL. Data included sex, age, plasma concentrations of free metanephrines, and genetic test results. For a subset of 89 children tested for PPGL, concentrations of 24‐h urinary fractionated metanephrines were also available. For patients with PPGL, data also included tumour location, size, catecholamine tumour phenotype, and presence of recurrent and/or metastatic disease.

**Results:**

Among children with PPGL, results of plasma metanephrines showed larger fold increases of normetanephrine above the upper cut‐off compared to the urinary metabolites (9.5‐fold vs 7.1‐fold, *p* < 0.001). Plasma metanephrines showed a diagnostic sensitivity of 92% and specificity of 96%, whereas for urinary metanephrines sensitivity and specificity of 92% and 91% respectively. Sub‐analysis of intra‐individual temporal measurements of metabolites showed that subsequent increases of plasma normetanephrine may be associated with early‐stage development of a noradrenergic PPGL.

**Conclusions:**

Plasma free and 24‐h urinary fractionated metanephrines are both reliable screening tests for PPGL in children and adolescents. The plasma panel may be useful for early detection of noradrenergic PPGL relevant for children tested within surveillance programs due to hereditary risk of noradrenergic tumours.

## 1 Introduction

Phaeochromocytomas and paragangliomas (PPGL) are rare but potentially lethal neuroendocrine tumours with an annual incidence of 0.5–2 per millions of children [1, 2]. PPGL exhibit numerous differences when they present in childhood compared to adults [3]. Many of these relate to the higher prevalence of hereditary disease in paediatric than adult patients [4]. Indeed, approximately 70%–80% of PPGL diagnosed in children are associated with germline pathogenic variants (PVs) of specific tumour‐susceptibility genes [4]. These can be divided into two main cluster groups: cluster 1 PVs result in the activation of the hypoxia signaling pathways and include those in the von Hippel‐Lindau (*VHL*) suppressor gene, or the succinate dehydrogenase complex *(SDHA, SDHB, SDHC, and SDHD)* genes [5]. The majority of the hereditary PPGL in childhood are Cluster 1 tumours and produce mainly norepinephrine. The second Cluster group includes tumours due to PVs that activate kinase signaling pathways such as those in *RET*, and *NF1*, represent approximately 4% of the hereditary PPGL, and produce mainly epinephrine [4, 6, 7].

The paediatric PPGL guidelines indicate plasma free or 24‐h urinary fractionated normetanephrine and metanephrine as the screening test of choice [8]. Importantly, data from the prospective clinical PMT‐trial showed that plasma free metanephrines offer superior diagnostic performance than the urinary metabolites for patients at high risk [9]. However, like almost all previous studies, the PMT‐trial was focused mainly on adult patients. Only a few reports focused on small paediatric cohorts, and showed that metanephrines, either in plasma or urine, demonstrate adequate diagnostic sensitivity for diagnosis of PPGL in children [10, 11].

An important component of precision medicine in paediatrics is the identification of children at risk of PPGL because of germline PVs through genetic testing. Surveillance programs are offered to families of all children with hereditary disease, as early detection of PPGL, especially in patients with *SDHB* pathogenic variants, is likely to minimize if not avoid metastatic progression [12]. Indeed, early tumour detection during surveillance programs is feasible, as PPGL are typically slow growing with a volume doubling time of 5–7 years [13, 14]. This is also reflected by time dependent increases in plasma concentrations of metanephrines [15]. Changes in metabolites over time during surveillance may signal development of a PPGL before concentrations become high enough to confirm disease [11].

Based on the above considerations, the main aim of this study was to examine whether measurements of plasma free and 24‐h urinary fractionated metanephrines provide reliable and robust tests for diagnosis of PPGL in children and adolescents. A secondary aim was to investigate whether intra‐individual temporal subsequent changes of plasma free metanephrines can signal early development of a PPGL in children tested during surveillance programs.

## 2 Methods

### 2.1 Patients

This study included retrospective data from 1998 to 2020 from 138 children and adolescents, 5 to 18 years, 64 with and 74 without PPGL, enrolled under the PMT clinical protocol (https://pmt-study.pressor.org/) in the University Hospital in Dresden, and the 00‐CH‐0093 protocol in the National Institutes of Health, Bethesda, USA. Verbal ascent and written informed consent were provided by all parents and their children respectively. Collected clinical data included date of study inclusion, age, sex, reason for testing, age of initial tumour diagnosis, previous history of PPGL, presence of multifocal, recurrent and/or metastatic disease and initial tumour(s) size. Data also included measurements of plasma free normetanephrine and metanephrine, whereas for a subset of 89 patients tested for PPGL measurements of 24‐h urinary fractionated normetanephrine and metanephrine were also available.

Exclusion of PPGL was established according to the following criteria: 1. negative biochemical test results (and/or imaging) at follow‐up a year after inclusion in the study; 2. An alternative diagnosis for a resected tumour histopathologically confirmed or; 3. Negative imaging studies and/or negative follow‐up confirmatory tests (e.g. clonidine suppression test) in patients with positive test results. Confirmation of PPGL required histopathological examination of surgically resected or biopsied tissue other than head and neck, or a diagnosis of inoperable metastasized disease based on functional imaging evidence of metastatic lesions. Metastatic disease was defined as the presence of metastases in non‐chromaffin tissues distant from the primary tumour (e.g. lungs, liver, bones, and lymph nodes) [16]. Synchronous metastases were defined by the presence of metastases within 1 year of diagnosis of the primary tumour. Recurrent disease was defined as the presence of loco‐regional recurrence and/or new tumour(s).

For nine children (cases 1–9) enrolled in surveillance programs, at least three subsequent measurements of plasma free normetanephrine and metanephrine were reported within intervals of five to 15 months and over a period of two to 7 years. Among them, in four children PPGL was excluded, whereas five were diagnosed with PPGL during the last reported biochemical work‐up (Appendix A1).

### 2.2 Genetic Testing

Genetic testing was performed in all children tested for PPGL. Testing for germline PVs of *VHL, RET, SDHB, SDHC, SDHD, SDHA, MAX*, and *TMEM127* was performed using Sanger sequencing and/or NGS, and multiplex ligation‐dependent probe amplification or custom array CGH for deletion detection. For *NF1*, the diagnosis was based on clinical manifestations according to established criteria [17].

### 2.3 Biochemical Measurements

Measurements of plasma free and 24‐h urinary fractionated metanephrines were performed using liquid chromatography with electrochemical detection [18] or liquid chromatography with tandem mass spectrometry [19]. Age‐adjusted upper cut‐offs for plasma normetanephrine and upper cut‐offs for metanephrine were as previously published [8]. Upper cut‐offs for 24‐h urinary normetanephrine and metanephrine were as provided by the laboratory of Mayo Clinic, Rochester, Minnesota (https://pediatric.testcatalog.org/show/METAF). Blood samples were collected after overnight fasting and at least 20 min of supine rest in heparinized tubes and kept on ice until centrifugation to separate plasma. Urine was collected over 24 h and returned to the study center, where urine volumes were determined and samples were aliquoted. 24‐h urinary fractionated metanephrines were measured after acidic hydrolysis. This step was necessary to convert sulfate‐conjugated metabolites into free form to allow measurement of combined free plus deconjugated metabolites. Designation of an adrenergic biochemical phenotype required an elevated plasma concentration of metanephrine above the upper cut‐off and a tumour‐derived increase of metanephrine of more than 5% of the combined increases of normetanephrine and metanephrine over the upper reference limits. All other tumours were defined as noradrenergic/dopaminergic.

### 2.4 Data Analysis

Continuous variables are shown as geometric means with confidence intervals of means. Comparison of continuous parameters was performed with Mann–Whitney *U* test. Categorical parameters were analyzed using chi‐square test. Diagnostic sensitivity was defined as the percentage of true positive results among both true positive and false negative results. Diagnostic specificity was defined as the percentage of true negative results among both true negative and false positive results. Subsequent increases of metabolites were defined as at least two consequent fold increases (%) of the metabolites in a 6‐month interval. Statistical analysis was performed using IBM SPSS Statistics. *p* < 0.05 was considered statistically significant.

## 3 Results

### 3.1 Patient Characteristics

Among 138 children and adolescents included in the study, 74 without and 64 with PPGL (Table 1). There were no differences in age or sex between the two groups. The majority of children in both groups (78% without and 69% with PPGL) were tested as part of surveillance programs due to hereditary risk and/or previous history of PPGL. As expected, 70% and 84% of children without and with PPGL respectively presented with PVs in Cluster 1 genes, whereas only 4% and 3% respectively with PVs in Cluster 2 genes. Genetic testing was negative in 26% and 13% of children without and with PPGL respectively. All children with negative genetic test results were initially tested due to signs and symptoms of catecholamine excess. As expected, plasma and urinary concentrations of normetanephrine were higher in children with than those without PPGL (*p* < 0.001). Furthermore, 34% (22/64) of all children diagnosed with PPGL were with metastatic disease. Among them, 36% (8/22) were diagnosed with synchronous metastases, while the remaining 64% (14/22) with metachronous. Importantly, 77% (17/22) of all children with metastatic disease harboured PVs in Cluster 1 genes.

**Table 1:** Characteristics of children and adolescents excluded or diagnosed with PPGL in the entire cohort.

	Patients without PPGL	Patients with PPGL	*p* value
Number	54% (74/138)	46% (64/138)	
Age	13 (10‐19)	13 (10‐18)	0.447
Sex (female)	45% (33/74)	42% (27/64)	0.345
Reason for testing			
Signs and Symptoms	22% (16/74)	31% (20/64)	0.084
Hereditary Risk/Previous PPGL	78% (58/74)	69% (44/64)	
Hereditary risk	*59% (44/74)*	*36% (23/64)*	
Previous history of PPGL	*19% (14/74)*	*33% (21/64)*	
Genetic testing			
Cluster 1	70% (52/74)	84% (54/64)	0.083
*VHL*	38	29	
*SDHB*	12	21	
*SDHC*	—	1	
*SDHD*	2	1	
*HIF2A*	—	2	
Cluster 2	4% (3/74)	3% (2/64)	
*RET*	3	1	
*NF1*	—	1	
Negative	26% (19/74)	13% (8/64)*	
Plasma free metabolites (pg/mL)			
Normetanephrine	48 (29–79)	670 (207–2167)	< 0.001
Metanephrine	30 (18–50)	31 (13–71)	0.776
24‐h urinary fractionated metabolites (μg/day)**			
Normetanephrine	172 (100–299)	1472 (450–4814)	0.001
Metanephrine	75 (38–150)	97 (43–219)	0.304

*Note:* Continuous parameters are shown as geometric means and CI.

* A child diagnosed with PPGL and with negative genetic testing had EPAS (HIF2A) somatic variant.

** Urine samples were available for only 89 children with and without PPGL.

Children diagnosed with PPGL presented more often with noradrenergic tumours, mainly due to the higher prevalence of Cluster 1 PPGL. In particular, children with Cluster 1 PPGL presented with increased concentrations of plasma free normetanephrine, whereas concentrations of plasma free metanephrine remained within the normal range (Table 2). In contrast, the two children with Cluster 2 PPGL presented with both elevated concentrations of plasma free metanephrine and increases of metanephrine larger than 5% of the sum of normetanephrine and metanephrine (Table 2). We observed similar correlations between the genetic clusters and the 24‐h urinary fractionated metabolites. Importantly, plasma free normetanephrine showed larger fold increases above the upper cut‐off value, compared to 24‐h urinary fractionated normetanephrine (9.5‐fold vs 7.1‐fold, *p* < 0.001). In contrast, plasma free and 24‐h urinary fractionated metanephrine showed overall no increases above upper cut‐offs in patients with PPGL.

**Table 2:** Biochemical phenotype of children with hereditary PPGL.

	Children wih Cluster 1 PPGL	Chilrden with Cluster 2 PPGL*
Biochemical test results		
Plasma free metabolites (pg/mL)		
*Normetanephrine*	528 (365–765)	808 (‐)
*Metanephrine*	28 (23–35)	183 (‐)
24‐h urinary fractionated metabolites (μg/day)*		
*Normetanephrine*	1114 (739–1679)	6662 (‐)
*Metanephrine*	76 (61–95)	1884 (‐)

*Note:* Continuous parameters are shown as geometric means and CI.

* Only two cases with confirmed PPGL and only one of them with available urinary panel.

### 3.2 Diagnostic Performance of Plasma Free and 24‐h Urinary Fractionated Metanephrines

Among 64 children with PPGL in the entire cohort, all but five presented with positive plasma biochemical test results, so that the plasma panel reached a sensitivity of 92%. The diagnostic specificity of the plasma panel was similarly high and reached 96% (Table S1). Specifically, among 74 children without PPGL, all but three presented with negative plasma biochemical test results.

Regarding the subgroup of 89 children, where both plasma and 24‐h urinary fractionated metanephrines were available (Table S2), 36 children were diagnosed with PPGL, and 34 presented with positive plasma biochemical tests compared to 33 children for urine measurements. Among 53 children without PPGL, all presented with negative plasma biochemical tests, whereas five children presented with false positive urinary test results. Thus, the plasma panel presented with a sensitivity of 94%, whereas the urinary panel 92% (Table 3). Diagnostic specificity for the plasma panel was 100%, whereas for the urinary panel 91%. Characteristics of all cases with false negative or false positive biochemical test results are described in the Appendix A1.

**Table 3:** Diagnostic performance of plasma free and 24‐h urinary fractionated metanephrines in the subgroup of 89 children tested for PPGL.

	Plasma free metanephrines	24‐h urinary fractionated metanephrines
**Sensitivity% (95%CI)** True positives / (True positives + false negatives)	94% (81%–99%) 34/36	92% (83%–100%) 33/36
**Specificity% (95%CI)** True negatives / (True negatives + false positives)	100% (93%–100%) 53/53	91% (83%–98%) 48/53

Abbreviation: CI, Confidence interval; n/N, correctly classified cases (sensitivity: true positives, specificity: true negatives)/total number of cases (with or without disease).

### 3.3 Subsequent Increases of Plasma Free Normetanephrine and Metanephrine Concentrations

**Figure 1:**
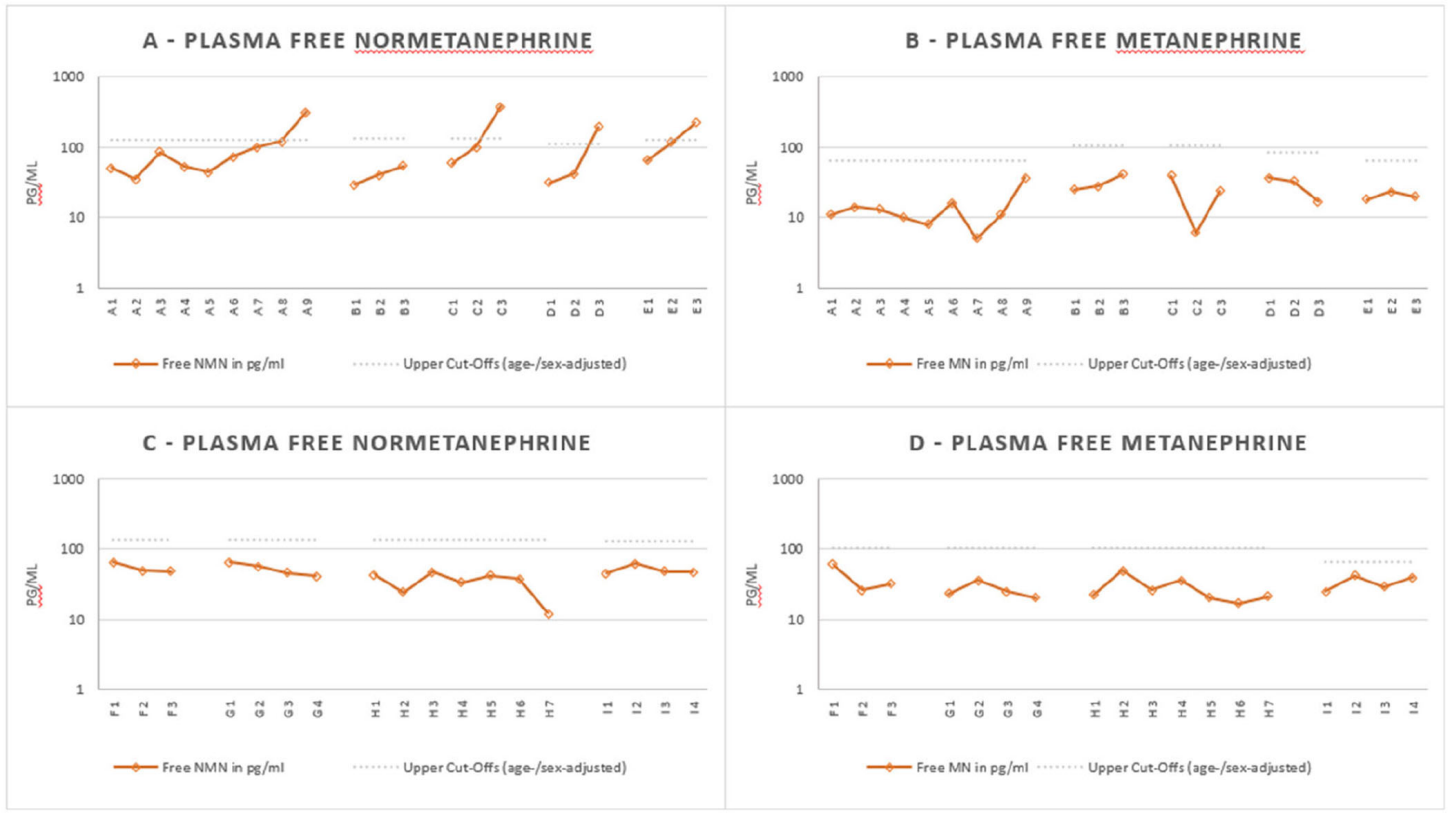
Temporal subsequent measurements of plasma free normetanephrine and metanephrine in five children with (Panels A, B) and four without PPGL (Panels C, D) during surveillance programs. For patients with PPGL, diagnosis was established during the time of the last biochemical work‐up. A – PLASMA FREE NORMETANEPHRINE. B – PLASMA FREE METANEPHRINE. C ‐ PLASMA FREE NORMETANEPHRINE. D ‐ PLASMA FREE METANEPHRINE.

Multiple measurements of metanephrines were available for nine children tested for PPGL, five with PPGL and four without, and only in plasma samples. Time points for measurements were chosen according to the individual risk of the patient with screening intervals of five to 15 months. For the analysis of subsequent increases, at least three measurements were required, and only in children that were either followed up due to a previous history of non‐metastatic PPGL or regularly screened due to hereditary risk. Among children with PPGL, diagnosis was established during the time of the last biochemical work‐up. Definite diagnosis was either by positive biochemical test (cases 1, 3–5) or imaging studies (case 2). Four presented with PVs of Cluster 1 genes and one child had negative genetic testing (case 1). Mean 6‐month percentage increases of plasma free normetanephrine were significantly higher in the five children diagnosed with PPGL than in the four without the tumour (46.6% vs 5.5%, *p*< 0.001), whereas for plasma free metanephrine they did not differ. Subsequent increases in normetanephrine (Figure 1A, Table S3) during the last biochemical test before definite tumour diagnosis were observed in all children with PPGL, without any significant subsequent increases for metanephrine (Figure 1B, Table S3). None of the children without PPGL presented with subsequent increases of either plasma free normetanephrine (Figure 1C,D, Table S4) or metanephrine. Further characteristics of all nine cases are described in detail in the Appendix A1.

## 4 Discussion

This study shows that both plasma free and 24‐h urinary fractionated metanephrines are reliable and robust tests for diagnosis of PPGL in children and adolescents. In addition, the plasma panel may be more useful than the urinary panel for the early detection of PPGL in children under surveillance programs, as reflected by the larger fold increases above the upper cut‐offs of plasma free compared to the 24‐h urinary fractionated normetanephrine. Importantly, temporal measurements of plasma metabolites in nine children tested for PPGL during surveillance showed that subsequent increases in plasma free normetanephrine can signal early‐stage development of a noradrenergic PPGL.

Diagnosis of PPGL in children has historically involved urinary tests of catecholamine excess [13, 20, 21]. However, for young children, collections of 24‐h urine may be troublesome. Thus, blood sampling is increasingly used in paediatric settings. In our study, we showed that plasma free and 24‐h urinary fractionated metanephrines both offer reliable screening tests for children tested with PPGL.

Despite the similar diagnostic performance of the two tests, tumour biochemical signal as reflected by fold increases of normetanephrine above the upper cut‐offs was stronger for the plasma than the urinary test. This is mainly attributed to the fact that plasma free metabolites are mainly formed within the chromaffin cells by the metabolism of catecholamines by COMT [22], whereas measurements of urinary metabolites are performed after hydrolysis step and include sulfate‐conjugated metabolites mainly formed in the gastrointestinal tract by the enzyme SULTA3 [23]. Another important finding was that subsequent increases of normetanephrine concentrations may provide a biochemical signal of early tumour development that could be used to guide individualized surveillance strategies (e.g. justify performance of imaging studies or shorter intervals of follow‐up) in daily clinical practice.

Another advantage of the plasma over the urinary (spot or 24‐h) metabolite test is the opportunity to add free methoxytyramine to the plasma panel. Indeed, it is well established that addition of this metabolite to the plasma test not only allows the identification of a small but significant proportion of patients with dopaminergic tumours [24], but for children with additional increases in other metabolites, that pattern can be useful to better predict the presence of a catecholamine‐producing tumour and from that move forward to further confirm disease by imaging studies [9, 25, 26]. Finally, a dopaminergic phenotype raises the likelihood of metastatic disease. In particular, in the PMT trial among patients with highly increased levels of methoxytyramine, the posttest probability of metastatic disease was 85% [25]. It should be appreciated, though, that in urine methoxytyramine mainly reflects the clearance and metabolism of l‐Dopa, and its value for the identification of dopamine‐producing tumours is limited [27, 28].

The benefits of the plasma panel are particularly relevant for children under surveillance due to PVs of Cluster 1 genes, where maximally sensitive tests to detect noradrenergic/dopaminergic PPGL at an early stage of development, when still too small to produce sufficient biochemical signal and thus positive test results, are needed to minimize the risk of metastatic disease [12]. Indeed, consistent with previous findings [4, 6], we demonstrate that children with Cluster 1 PVs present more often with noradrenergic tumours, characterized by increased plasma free normetanephrine, but relative lack of increased plasma free metanephrine. Importantly, this group of patients exhibits a higher prevalence of metastatic disease [4, 6], highlighting the importance of early tumour detection through maximally sensitive biochemical tests. Nevertheless, for the rare group of children at surveillance due to PVs of Cluster 2 genes, who are at risk of developing adrenergic tumours [29] with very low metastatic potential, urinary testing is an equally good alternative, and the choice of urine vs plasma test could be guided by availability, experience, and practical considerations.

Blood sampling should be performed with adherence to specific pre‐analytical precautions, including sampling in a warm relaxed environment after at least 20 min of supine rest, as activation of the sympathetic nervous system by the upright posture increases plasma concentrations of normetanephrine, leading to higher rates of false positive results [30]. In addition, distress‐triggered sympathoadrenal activation can be further reduced by sampling through an intravenous cannula rather than by direct venepuncture [8, 30]. Importantly, when plasma free methoxytyramine is added to the biochemical test, overnight fast, if possible, should be suggested to parents [31]. If the pre‐analytical precautions cannot be followed, then the diagnostic performance of the plasma metabolites can be significantly reduced [30, 32] and urinary tests should be preferred. Apart from 24‐h, first morning spot urine could be an alternative option [33, 34] for the exclusion of PPGL among young children with a low risk of PPGL (e.g., children tested only due to signs and symptoms) and needle phobia, but additional prospective studies are needed to validate the diagnostic value of first morning spot urine in children.

Our study has limitations. First, our findings derive from the analysis of retrospective paediatric cohort with possible referral and temporal bias and limited number of children included. A prospective population‐based clinical trial is required to assess the diagnostic performance of the two biochemical tests. In addition, although in plasma we assessed the free metabolites, in 24‐h urinary collections only fractionated and not free metanephrines were available. Importantly, difficulties in the collection of 24‐h urine, especially among young children, may have underestimated the test's diagnostic performance. Finally, temporal measurements of plasma free metanephrines were reported only in plasma, and only in nine children, so that future studies in larger paediatric cohorts are needed to validate our findings.

Despite the aforementioned limitations, our study includes a well characterized cohort of children tested for PPGL and shows that both plasma free and 24‐h urinary fractionated metanephrines are reliable screening tests. Importantly, our study provides preliminary elements that the plasma panel may be a useful test for the detection of early stage noradrenergic/dopaminergic PPGL in children under surveillance programs either due to PVs of Cluster 1 genes or previous history of noradrenergic tumours. These findings are expected to guide further individualized management and follow‐up strategies of children tested for PPGL.

## Conflicts of Interest

The authors declare no conflicts of interest.

## Supporting information

Supplemental Appendix.
