# Supplementary material for: Biochemical Diagnosis of Phaeochromocytoma and Paraganglioma in Children and Adolescents: A Retrospective Cohort Study
Source: Clin Endocrinol (Oxf). 2025 Aug 21;103(6):787–94. doi: 10.1111/cen.70025 (PMC12583316; doi:10.1111/cen.70025)
Supplement: Supplementary file 1 — Supplemental Appendix. [file CEN-103-787-s001.docx]

**Supplemental Appendix**

**Biochemical diagnosis of phaeochromocytoma and paraganglioma in children and adolescents: a retrospective cohort study**

Kristin Potthoff^1^, Tamara Prodanov^2^, Lara M. Knigge^1^, Angela Hübner^3^, Stefan Bornstein^1^, Jacques Lenders^4^, Karel Pacak^2^, Graeme Eisenhofer^1^, Christina Pamporaki^1^

^1^Department of Medicine ΙΙI, University Hospital Carl Gustav Carus at the TU Dresden, Dresden, Germany

^2^National Institutes of Health (NIH), Bethesda, USA

^3^Department of Paediatrics, University Hospital Carl Gustav Carus at the TU Dresden, Dresden, Germany

^4^Department of Internal Medicine, Radboud University Medical Centre, Nijmegen, the Netherlands

**Supplementary results**

Supplementary Table 1 shows the diagnostic performance of plasma free normetanephrine and metanephrine in the entire cohort of 138 children tested for PPGL.

| **Supplementary Table 1**: Diagnostic performance of plasma free normetanephrine and metanephrine in the entire cohort | |
| --- | --- |
| **Sensitivity% (95%CI)**  Count n/N | 92% (86%-99%)  59/64 |
| **Specificity% (95%CI)**  Count n/N | 96% (91%-100%)  71/74 |

*CI: Confidence interval; n/N = correctly classified cases (sensitivity: true positives, specificity: true negatives) / total number of cases (with, without disease)*

**Characteristics of patients in the subgroup of 89 children with both plasma free and 24-hour urinary fractionated metabolites available**

Among 89 paediatric patients with both plasma and 24-urinary test results available, 53 were excluded and 36 were diagnosed with PPGL. Children diagnosed with PPGL were younger (P=0.025) than those without the disease. As expected, the majority of children tested with PPGL (67%) were under surveillance and follow-up programs mainly due to PVs in Cluster 1 susceptibility genes. Consequently, children with PPGL presented mainly with noradrenergic tumours and elevations in plasma and 24-hour urinary concentrations of normetanephrine (Supplementary Table 2).

| **Supplementary Table 2**: Characteristics of children with and without PPGL in a subgroup of 89 patients with both plasma free and 24-hour urinary fractionated metanephrines available | | | |
| --- | --- | --- | --- |
|  | **Without PPGL** | **With PPGL** | **P Value** |
| **Number** | 60% (53/89) | 40% (36/89) |  |
| **Age** | 15 (2-18) | 14 (6-18) | 0.025 |
| **Sex (female)** | 49% (26/53) | 42% (15/36) | 0.381 |
| **Reason for testing** |  |  |  |
| Signs and Symptoms | 13% (7/53) | 33% (12/36) | 0.023 |
| Hereditary Risk/Previous PPGL | 87% (46/53) | 67% (24/36) |  |
| **Genetic Testing** |  |  |  |
| Cluster 1 | 81% (43/53) | 83% (30/36) | 0.560 |
| Cluster 2 | 2% (1/53) | 3% (1/36) |  |
| Negative | 17% (9/53) | 14% (5/36) |  |
| **Plasma free metabolites (pg/mL)** |  |  |  |
| Normetanephrine | 47 (28-82) | 651 (220-1921) | 0.001 |
| Metanephrine | 27 (16-46) | 37 (17-81) | 0.102 |
| **24-hour urinary fractionated metabolites (μg/day)** |  |  |  |
| Normetanephrine | 172 (100-299) | 1472 (450-4814) | 0.001 |
| Metanephrine | 75 (38-150) | 97 (43-219) | 0.304 |

*Continuous parameters are shown as geometric means and CI*

**Characteristics of patients with PPGL and false negative biochemical test results**

Among 36 children with PPGL and both plasma and 24-hour urinary test results available, two presented with false negative 24-hour urinary fractionated metanephrines but positive plasma metabolites, one presented with both false negative plasma and urinary metabolites, whereas one patient presented with negative plasma free metanephrines but positive urinary metabolites. In particular, the first case was an 18-year-old patient tested due to previous history of PPGL and known *SDHB* pathogenic variant (PV). The initial tumour, a 12x11x7 cm right pheochromocytoma, was diagnosed at the age of 14. At study inclusion, the patient presented with elevated plasma free normetanephrine concentrations but normal urinary metabolites. Imaging studies revealed a paraganglioma as well as bone and liver metastases. The second case was a 14-year-old boy initially tested due to abdominal pain. Biochemical testing showed significant elevations of plasma free normetanephrine but normal urinary metabolites. Anatomical imaging studies revealed a right paraganglioma of 7x4x5 cm, whereas functional imaging also detected bone and liver metastases. Genetic testing was negative. The third case was a 16-year-old girl with known *SDHB* PV who was diagnosed with a retroperitoneal paraganglioma and metastatic bone lesions. Nevertheless, both plasma free and 24-hour urinary metanephrines were normal at the time of diagnosis. Finally, there was a 13-year-old female with symptoms e.g. headache and tremor. She underwent biochemical testing and imaging, where a head and neck paraganglioma was found. The levels of plasma free metanephrines were normal, whereas 24-hour urinary metabolites were elevated. Genetic testing was positive for *SDHB* PV.

Among the 64 children with PPGL from the entire cohort, there were three additional cases with false negative results for plasma free metanephrines, apart from the aforementioned 16-year-old and 13-year-old girls. The first case was a 13-year-old boy with a nonfunctional bladder paraganglioma and negative plasma test results. Genetic testing was positive for *SDHB* PV. The second case was a 9-year-old boy tested due to signs and symptoms (abdominal pain, vomiting). The child was diagnosed with a retroperitoneal paraganglioma and metastatic bone lesions. Genetic test results were positive for *SDHB* PV. The final case was a 13-year-old female with negative plasma free metanephrines, diagnosed with a head and neck and a superior mediastinal paraganglioma. Genetic testing was positive for *SDHD* PV.

**Characteristics of patients without PPGL and false positive biochemical test results**

Among the 53 children without PPGL with both plasma and 24-hour urinary test results available, five cases presented with false positive urinary test results. The first case was an 11-year-old female with *SUCGL2* PV who presented with recurrent symptoms of catecholamine excess a year after surgical removal of a phaeochromocytoma. Biochemical testing for plasma free metanephrines was negative but positive for 24-hour urinary fractionated normetanephrine levels. Imaging studies followed, a tumour though was not detected. The second case was an 8-year-old girl with known *RET* PV and tested under surveillance program. Biochemical testing revealed elevated 24-hour urinary fractionated normetanephrine and metanephrine levels, whereas plasma free metanephrines were normal. Imaging studies followed, and no tumour was found. Finally, three children with *VHL* PVs – a 13-year-old boy, an 11-year-old girl and a 15-year-old girl – under surveillance programs, presented with positive 24-hour urinary fractionated normetanephrine, but normal plasma free metanephrines. PPGL was excluded by imaging studies.

Among the 74 children without PPGL from the entire cohort, three presented with false positive plasma free metanephrines. The first was a 2-year-old boy with cystinosis who underwent diagnostic work-up for secondary hypertension. He presented with elevated plasma free normetanephrine levels. Genetic testing was negative, and no tumour was detected on MRI. The second was a 5-year-old girl with a known *SDHB* PV and signs and symptoms of catecholamine excess (episodes of hot flashes). Plasma free normetanephrine levels were elevated. Nevertheless, no tumour was detected on MRI. The third case was an 18-year-old male, tested due to unexplained hypertensive crisis. Biochemical testing showed elevated levels of plasma free normetanephrine. There was no family history of the tumour. PPGL was excluded by negative biochemistry at follow-up. 24-hour urinary metabolite test results were not available for these cases.

**Characteristics of children with temporal subsequent measurements of plasma free metanephrines during long term surveillance/follow-up programs**

Among children with confirmed PPGL during the last follow up, **case 1** was a 13-year-old girl. She first presented in August 2006 in the clinic with paroxysmal hypertension, headaches, sweatiness and palpitations. A 7x5x4.5 cm right adrenal tumour was diagnosed and the patient was operated in September 2006. Genetic testing revealed no germline PVs in PPGL susceptibility genes. Supplementary Table 3 includes temporal subsequent measurements of plasma free metanephrines after operation of the primary tumour. Seven years later, and during the last follow up, a recurrent adrenal tumour with metastatic disease was diagnosed. The second patient (**case 2**) was an 11-year-old boy, initially tested for PPGL in 2017 due to abdominal pain and vomiting. A left adrenal phaeochromocytoma was diagnosed with dimensions 4.8x4.5x3.6 cm and the patient was operated in the same year. Genetic testing revealed a *SDHB* PV. Postoperatively the patient remained under regular follow up with subsequent measurements of plasma free metanephrines (Supplementary Table 3). In March 2019 and despite negative biochemical testing, imaging studies revealed a bladder PPGL, which was histopathologically confirmed after surgery in the same year. **Case 3**, was an 11-year-old boy tested under a *VHL* screening program. In March 2001 the last biochemical testing (Supplementary Table 3) was positive and the imaging studies revealed a left phaeochromocytoma of 3x3x.5 cm without any signs of metastases. **Case 4**, was a 12-year-old girl tested under a *VHL* screening program and during the last biochemical testing was diagnosed with a 1.2.x1x1 cm left phaeochromocytoma without any signs of metastases. **Case 5** was a 16-year-old male patient tested during a *VHL* screening program and was diagnosed during the last biochemical work up with bilateral phaeochromocytoma of 3.5 and 1.2 cm respectively.

Among children excluded for PPGL, **case 1** was a 10-year-old boy with known *SDHB* pathogenic variant and previous history of an abdominal PGL of 3.9x2.7x2.2cm that was operated in 2011. Follow-up from 2012 to 2013 showed negative biochemical testing (Supplementary Table 4) and imaging studies showed no suspicion of recurrent PPGL or metastatic disease. **Case 2**, was an 11-year-old boy who was initially diagnosed in January 2010 with a right phaeochromocytoma of 3.8x2.9x1.5 cm and operated a month later. Genetic testing was negative for *SDHx* PV. The post-operative follow up from 2010 to 2012 was negative. **Case 3**, was an 8-year-old boy under follow up due to previous history of a left phaeochromocytoma of 6x4x3.5 cm, operated in 2010. Genetic testing revealed *SDHD* from the paternal side. Supplementary Table 4 shows that during the follow up from 2012 to 2019 biochemistry was negative and there was no sign of recurrent or metastatic disease. Finally, **case 4** was a 17-year-old boy under surveillance due to *VHL* PV. Clinical and biochemical screening between the years 1998-2000 was negative.

| **Supplementary Table 3:** Subsequent measurements of plasma free metanephrines in children under long term surveillance/follow-up programs with diagnosis of primary or recurrent PPGL at the time of the last follow up. | | | | | | | | | |
| --- | --- | --- | --- | --- | --- | --- | --- | --- | --- |
| **Case 1** | **A1** | **A2** | **A3** | **A4** | **A5** | **A6** | **A7** | **A8** | **A9** |
| *Date of Blood Sampling* | 27.10.2006 | 04.05.2007 | 05.11.2007 | 02.01.2009 | 08.03.2010 | 03.01.2012 | 29.07.2012 | 15.01.2013 | 29.05.2013 |
| *Plasma Free Normetanephrine(pg/ml)* | 50 | 35 | 85 | 53 | 44 | 73 | 100 | 120 | 315 |
| *Plasma Free Metanephrine (pg/ml)* | 11 | 14 | 13 | 10 | 8 | 16 | 5 | 11 | 37 |
| **Case 2** | **B1** | **B2** | **B3** |  |  |  |  |  |  |
| *Date of Blood Sampling* | 16.10.2017 | 30.05.2018 | 11.03.2019 |  |  |  |  |  |  |
| *Plasma Free Normetanephrine(pg/ml)* | 29 | 40 | 54 |  |  |  |  |  |  |
| *Plasma Free Metanephrine (pg/ml)* | 25 | 28 | 41 |  |  |  |  |  |  |
| **Case 3** | **C1** | **C2** | **C3** |  |  |  |  |  |  |
| *Date of Blood Sampling* | 26.01.1999 | 08.12.1999 | 05.03.2001 |  |  |  |  |  |  |
| *Plasma Free Normetanephrine (pg/ml)* | 59 | 99 | 372 |  |  |  |  |  |  |
| *Plasma Free Metanephrine (pg/ml)* | 39 | 6 | 24 |  |  |  |  |  |  |
| **Case 4** | **D1** | **D2** | **D3** |  |  |  |  |  |  |
| *Date of Blood Sampling* | 19.05.1999 | 22.09.2000 | 09.02.2004 |  |  |  |  |  |  |
| *Plasma Free Normetanephrine (pg/ml)* | 31 | 42 | 195 |  |  |  |  |  |  |
| *Plasma Free Metanephrine (pg/ml)* | 37 | 33 | 17 |  |  |  |  |  |  |
| **Case 5** | **E1** | **E2** | **E3** |  |  |  |  |  |  |
| *Date of Blood Sampling* | 19.08.1998 | 03.08.1999 | 01.08.2000 |  |  |  |  |  |  |
| *Plasma Free Normetanephrine (pg/ml)* | 65 | 116 | 220 |  |  |  |  |  |  |
| *Plasma Free Metanephrine (pg/ml)* | 18 | 23 | 20 |  |  |  |  |  |  |

| **Supplementary Table 4:** Subsequent measurements of plasma free metanephrines in children under long term surveillance/follow-up programs without diagnosis of primary or recurrent PPGL at the time of the last follow up . | | | | | | | |
| --- | --- | --- | --- | --- | --- | --- | --- |
| **Case 1** | **F1** | **F2** | **F3** |  |  |  |  |
| *Date of Blood Sampling* | 03.06.2012 | 02.01.2013 | 03.11.2013 |  |  |  |  |
| *Plasma Free Normetanephrine (pg/ml)* | 64 | 49 | 48 |  |  |  |  |
| *Plasma Free Metanephrine (pg/ml)* | 60 | 26 | 32 |  |  |  |  |
| **Case 2** | **G1** | **G2** | **G3** | **G4** |  |  |  |
| *Date of Blood Sampling* | 08.07.2010 | 31.10.2010 | 22.05.2011 | 05.02.2012 |  |  |  |
| *Plasma Free Normetanephrine (pg/ml)* | 65 | 57 | 46 | 41 |  |  |  |
| *Plasma Free Metanephrine (pg/ml)* | 23 | 36 | 25 | 20 |  |  |  |
| **Case 3** | **H1** | **H2** | **H3** | **H4** | **H5** | **H6** | **H7** |
| *Date of Blood Sampling* | 04.03.2012 | 04.11.2012 | 22.08.2013 | 20.02.2014 | 18.02.2015 | 28.07.2016 | 18.04.2019 |
| *Plasma Free Normetanephrine (pg/ml)* | 43 | 24 | 47 | 33 | 42 | 37 | 12 |
| *Plasma Free Metanephrine (pg/ml)* | 22 | 49 | 26 | 36 | 20 | 17 | 21 |
| **Case 4** | **I1** | **I2** | **I3** | **I4** |  |  |  |
| *Date of Blood Sampling* | 25.08.1998 | 09.03.1999 | 14.09.1999 | 11.07.2000 |  |  |  |
| *Plasma Free Normetanephrine (pg/ml)* | 45 | 62 | 48 | 47 |  |  |  |
| *Plasma Free Metanephrine (pg/ml)* | 25 | 42 | 29 | 39 |  |  |  |
